# Locomotor Adaptation versus Perceptual Adaptation when Stepping Over an Obstacle with a Height Illusion

**DOI:** 10.1371/journal.pone.0011544

**Published:** 2010-07-12

**Authors:** Christopher K. Rhea, Shirley Rietdyk, Jeffery M. Haddad

**Affiliations:** Department of Health and Kinesiology, Purdue University, West Lafayette, Indiana, United States of America; The University of Western Ontario, Canada

## Abstract

**Background:**

During locomotion, vision is used to perceive environmental obstacles that could potentially threaten stability; locomotor action is then modified to avoid these obstacles. Various factors such as lighting and texture can make these environmental obstacles appear larger or smaller than their actual size. It is unclear if gait is adapted based on the actual or perceived height of these environmental obstacles. The purposes of this study were to determine if visually guided action is scaled to visual perception, and to determine if task experience influenced how action is scaled to perception.

**Methodology/Principal Findings:**

Participants judged the height of two obstacles before and after stepping over each of them 50 times. An illusion made obstacle one appear larger than obstacle two, even though they were identical in size. The influence of task experience was examined by comparing the perception-action relationship during the first five obstacle crossings (1–5) with the last five obstacle crossings (46–50). In the first set of trials, obstacle one was perceived to be 2.0 cm larger than obstacle two and subjects stepped 2.7 cm higher over obstacle one. After walking over the obstacle 50 times, the toe elevation was not different between obstacles, but obstacle one was still perceived as 2.4 cm larger.

**Conclusions/Significance:**

There was evidence of locomotor adaptation, but no evidence of perceptual adaptation with experience. These findings add to research that demonstrates that while the motor system can be influenced by perception, it can also operate independent of perception.

## Introduction

The control of human locomotion is not trivial. Humans routinely traverse complex, obstacle-laden environments containing stairs, curbs and gaps. The ability to adapt gait to the constraints imposed by the environment requires individuals to scale their motor actions to the perceived dimensions of these environmental obstacles [1]–[5]. Previous research has shown that when stepping over a gap [2] or climbing stairs [3]–[5] people will make larger stepping movements when they perceive a large obstacle compared to when they perceive a small obstacle. These studies suggest that motor actions are scaled to the perceived rather than the actual dimensions of an obstacle. One interesting implication of these findings is that trips may be avoided if steps (or other obstacles) are made to look perceptually larger than their actual height since this would result in individuals adopting a larger and safer stepping height [5].

Interestingly, other studies have not found a link between perception and action. Specifically, when acting on an object with dimensions altered by an illusion, movements are scaled to the actual and not the perceptual size of the object. Thus, what is visually perceived does not influence action, suggesting a dissociation between perception and action [6]–[10].

The discrepancy in scaling between perception and action may be due to the differences in context across studies. For example, when lifting two equally weighted, but perceptually different objects, participants' actions are initially scaled to the illusory weight of the object [11]. However, after repeatedly lifting the objects, participants adapted their action to the veridical weight of the objects despite the fact that the size-weight illusion persisted [11]. This finding suggests that the perception-action relationship may change as a function of task experience. If similar observations are observed in a locomotor task, this will provide evidence that a common visuomotor system is acting for both upper-limb and lower-limb movements [9].

It is important to note that perception is typically assessed by asking participants to indicate if one object is different from another or to assign some value to an object characteristic, such as height. These assessments provide a measure of the participants' conscious perception, or what can be termed ‘explicit perceptual awareness’. Participants may not be directly conscious of other types of perception, such as the perception-for-action that resulted in subjects demonstrating the same behavior in the size-weight study despite explicitly indicating they were different [11]. In this study, the participants judged the height of an obstacle; this judgment was used to quantify perception. More specifically, explicit perceptual awareness was quantified.

The aims of this study were therefore to explore the scaling of visual perception to visually guided action during adaptive gait, and to determine the influence of task experience on the scaling. Participants judged the height an obstacle before and after stepping over it 50 times. To examine if the perception of obstacle height influenced the action of stepping over the obstacle, the height judgment was compared to toe elevation when stepping over the obstacle in the first and last five obstacle crossing trials. Previous research has suggested that motor adaptation can occur, but perception is robust [11], [12]. Accordingly, two hypotheses were formed: 1) an association between perception and action would be observed in early stepping trials, and 2) a dissociation between perception and action would be observed in later stepping trials. If both hypotheses are accepted, it would provide evidence of motor adaption without perceptual adaptation following experience acting on the obstacle.

## Methods

### Participants

Fifteen adults (6 males and 9 females) participated in the study (age: 24.6±4.3 yrs, height: 1.74±0.11 m, weight: 73.4±15.6 kg). All procedures were approved by the institutional review board at Purdue University and all participants signed an informed consent form.

### Experimental setup

The experiment was conducted in low light (0.1 lux), with one 40 watt incandescent light placed 3 m behind the start of the walkway. To further minimize environmental visual cues that may provide information regarding the height of the obstacle, the floor was covered with gray indoor/outdoor carpet and the walls were covered with white fabric.

Two obstacles were used, a full obstacle (entire surface of obstacle was visible) and a perimeter obstacle (only top and side edges were visible). Obstacles were 30 cm by 78 cm by 0.5 cm (height by width by depth), with L-brackets attached at the bottom to hold the obstacle upright. The obstacles were composed of masonite (painted black) and covered with glow-in-the-dark tape. The full obstacle was covered completely with glow-in-the-dark tape. A 6.4 mm wide outline of the top and side edges of the perimeter obstacle were covered with glow-in-the-dark tape. The differences between the obstacles made the full obstacle appear larger than the perimeter obstacle. Various obstacle height illusions were tested prior to the experiment. The manipulation that provided the largest perceived height difference was the full and perimeter obstacles in a low light setting. The identification of this perceptual difference *a priori* was necessary in order to evaluate any concurrent differences (or lack thereof) in the motor domain while performing the obstacle crossing task.

### Instrumentation

One infra-red emitting diode (IRED) was placed at the end of a wand (used to indicate judgment of obstacle height) and one IRED was placed at the top of each obstacle. Lower limb displacement was recorded with eight IREDs placed bilaterally on the toe, heel, ankle, and knee. IREDs were placed on the lateral aspect of the right leg and medial aspect of the left leg so that they would be viewed by one 3D position sensor (Optotrak, Northern Digital, Inc.) facing the right side of the subject. The data were collected at 100 Hz and filtered at 8 Hz with a 4th order zero-phase-shift low-pass Butterworth digital filter.

### Procedure

Participants were randomly assigned to receive either the full obstacle or the perimeter obstacle first; the obstacle presentation order was followed for three tasks. First, subjects estimated each obstacle's height (pre-action). Second, they stepped over each obstacle. Third, they estimated each obstacle's height again (post-action).

**Figure 1:**
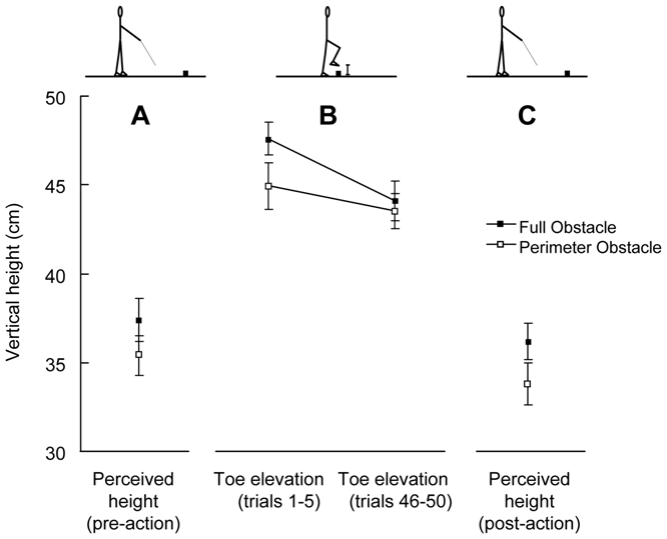
Mean data and standard error for perceived height and toe elevation. The pre-action perceived height (A), toe elevation (B), and post-action perceived height (C) conditions are shown. Both obstacles were 30 cm tall. The stick figures at the top of the figure depict the perceived height (A and C) and obstacle crossing (B) tasks. The full obstacle was judged to be taller than the perimeter obstacle in both the pre-action (A) and post-action (C) perceived height tasks (*p*>0.01). In the obstacle crossing task (B), an interaction was observed (*p* = 0.04), with a higher toe elevation for the full obstacle in trials 1–5, but no difference in toe elevation between obstacles in trials 46–50.

To evaluate perceived height, participants were given a handheld wand (dowel rod - 0.01 m diameter, 1.22 m length) and instructed to make contact at the base of a wall 1.5 m on their left side (see stick figure in top left of Figure 1). An obstacle was placed on the floor at a distance of 1.5 m in front of the participant. Participants were instructed to raise the wand vertically along the wall on the left, and to stop and hold the wand tip at the same height as the obstacle. The other obstacle was out of sight and no performance feedback was provided. Perceived height was assessed five consecutive times for each obstacle.

After the pre-action perception trials, participants walked down an 8 m walkway, stepped over a single obstacle (placed at 4 m), and progressed to the end of the walkway. This was repeated 50 consecutive times for each obstacle. No practice trials were performed. A total of 120 trials were recorded in the following order: (1) 10 pre-action perceived height trials (2 obstacles X 5 trials each), (2) 100 obstacle crossing trials (2 obstacles X 50 trials each) and (3) 10 post-action perceived height trials (2 obstacles X 5 trials each).

### Dependent Variables

The vertical distance between the marker on the wand and the floor was calculated to assess perceived obstacle height. The vertical distance between the toe marker (of the first foot that crossed the obstacle) and the floor when the toe IRED was directly over the obstacle was calculated to assess toe elevation. Perceived obstacle height was calculated for all perceived height trials; toe elevation was calculated for the first and last five obstacle crossing trials.

### Statistics

A two factor (obstacle type (full or perimeter) X trial block (pre- or post-action for perceived height; trials 1–5 or 46–50 for obstacle crossing) within-subjects repeated measures ANOVA was used for each dependent variable. Tukey post-hoc analyses were used where appropriate. Perceived height was not statistically compared to toe elevation as the goals are different in the two tasks. When crossing the obstacle, the goal is to lift the foot to clear the obstacle. When raising the wand, the goal is to match the height of the obstacle. The statistical design was used to examine the relation between the obstacles for each dependent variable in the initial and final conditions.

## Results

For perceived height, no trial block by obstacle type interaction was found (*F*_1,14_ = 0.08, *p* = 0.78). No main effect of trial block was observed (*F*_1,14_ = 2.01, *p* = 0.18). However, a main effect of obstacle type was detected (*F*_1,14_ = 14.34, *p*<0.01), with the full obstacle (36.8±5.3 cm) being perceived as taller than the perimeter obstacle (34.6±4.6 cm) (Figures 1A and 1C). Perceived height for both obstacles did not significantly decrease from pre- to post-action (*p*>0.05)

For toe elevation, a trial block by obstacle interaction was found (*F*_1,14_ = 5.47, *p* = 0.04). Post-hoc analyses revealed a higher toe elevation for the full obstacle compared to the perimeter obstacle in trials 1–5 (full: 47.6±3.5 cm, perimeter: 44.9±3.3 cm), but not in trials 46–50 (full: 44.1±4.1 cm, perimeter: 43.5±3.4 cm) (Figure 1B). Toe elevation for both obstacles significantly decreased from trials 1–5 to trials 46–50 (*p*<0.05). One trip (contact with the obstacle) was observed for all participants (<0.01% of all trials) and it was with the trail foot (second foot to step over the obstacle).

## Discussion

The findings demonstrate that while the motor system can be influenced by perception, it can also operate independent of the perceptual system. More specifically, the motor system can operate independent of explicit perceptual awareness. Although the veridical heights of the obstacles were identical, the full obstacle was perceived as 2.0 cm larger than the perimeter obstacle. In the early stepping trials, participants increased toe elevation by 2.7 cm, consistent with the perceived rather than veridical height of the obstacle. It is reasonable to assume that these perceptual differences led the participants to adopt the larger toe elevation when navigating over the full obstacle in the early stepping trials. However, toe elevation differences between obstacles were no longer observed in the later trials despite the fact that participants still perceived the full obstacle as larger than the perimeter obstacle. These findings suggest there is an association between perception and action in the early trials and a dissociation in the later trials. Therefore, it appears that motor adaptation can occur without concurrent adaptation of explicit perceptual awareness.

An association between perception and action during early trials has also been found in a stair-stepping task [5]. Elliott and colleagues [5] observed larger toe elevation when participants stepped onto a perceptually larger stair. However, Elliott et al. did not examine post-action perception, and only five trials per condition were collected [5], therefore no conclusions were made regarding how the association between perception and action changes with task experience.

Changes in the association between action and perception resulting from task experience have also been found in a lifting study [11]. In this study, two equally weighted objects of different size were perceived to weigh differently prior to lifting the objects. In the early lifting trials, an association between perception and action was observed: higher grip force was observed for the perceptually heavier object. In the late lifting trials, grip force was scaled to the veridical weight of the objects. However, the objects were still perceived to be of different weight after the lifting trials, suggesting that a dissociation emerged between perception and action with task experience. Similar findings from the current locomotor study and the lifting study strengthen the role of experience in the scaling between perception and action, and also supports the idea that a common visuomotor system is acting for both upper-limb and lower-limb movements [9].

Motor adaptation without concurrent perceptual adaptation is especially interesting as the participants never touched or handled the obstacle (apart from one toe-obstacle contact of a single subject), unlike the lifting study [11]. Apparently, information regarding obstacle height is being gathered; since there was no physical contact with the obstacle, the information must be visual. When stepping over obstacles, it is known that subjects monitor the position of the lower limb relative to the obstacle (as observed in the peripheral visual field) in an on-line manner [see review in 13]. The nervous system may have compared the expected position of limb relative to the obstacle versus the actual position. Due to the illusion, the actual position would be higher than expected, and this feedback was used to adjust subsequent stepping trials. These changes occurred without parallel adjustments in the cognitive factors that affect perception, supporting the idea that separate visual systems are responsible for the perception and the control of actions [6].

It is important to note that the manner in which perception and action were evaluated may have influenced the measures. Gibson [14] suggested that an evaluation of perception while the participant is seated or standing still (which is common practice) does not provide an accurate assessment. The optic flow available when moving contains higher order information that identifies invariant properties [14]. Information from these invariant properties can then be used to recover depth perception and direction of heading, among other things, and thus be used to control motion. According to Gibson, “We must perceive in order to move, but we must also move in order to perceive” [14, p. 223], suggesting there is a coupling between perception and action when navigating the environment. In this study, perception of obstacle height was evaluated while the participant was standing still, but the participant was moving toward the obstacle when presumably the perception-for-action was being formed in the gait trials. Although motor adaptation was observed, the perceived height relation between obstacles did not change. It is possible that visually guided action would have been scaled to visual perception independent of task experience if perception had been assessed while the person was moving through the environment. In addition, the participants did not receive the same amount of experience perceiving the obstacle height as they had acting on the obstacle. Previous research had shown that perception was robust to adaptation [12], and pilot studies indicated that the perception was not modified following multiple trials, so only five trials were examined to reduce tedium for the subjects. It is also possible that unconscious forms of perception are independent from explicit perceptual awareness, and these forms may have adapted with experience. This study design can only address changes in explicit perceptual awareness.

The manner in which actions are visually controlled depends on the type of visual information available. If visual information of the object is available throughout the action (termed online or closed-looped visual control), corrections to the movement pattern based on visual information can be made during the motion so that the end-point of the action reflects the actual (veridical) size of the object. This is observed even in the case of visual illusions, where a non-veridical perception of an object is present prior to movement, but online control nulls the effect of the illusion on the action, leading to a perception-action dissociation [9], [10], [15]. If online control is not available to correct the movement pattern (termed open-loop visual control), then a perception-action association is typically observed (i.e. action is scaled to perception) [9], [10], [15]. In this experiment, online visual information was available throughout all tasks. However, lower visibility due to low light decreased the visual richness of the environment, resulting in behavior similar to that observed with open-loop visual control [9], [10], [15]. A shift in behavior due to task experience was potentially due to participants directing their attention to relevant visual information (e.g. horizon line specified by eye height, texture gradient, corner of the room, etc.) that could have been used to visually guide their action in an online manner and null the effect of the perceptual manipulation on action. Gibson termed this process *education of attention* [14]. When the visual richness of the environment is decreased (e.g. low light), it may take task experience to appropriately direct visual attention.

In summary, the results support the hypothesis that the scaling between action and perception is dependent on task experience. The motor system demonstrated adaptation to the illusion, but the perceptual system did not adapt. More specifically, explicit perceptual awareness did not adapt. These findings add to research that demonstrates that while the motor system can be influenced by explicit perception, it can also operate independent of explicit perceptual awareness.
